# MEGAN Community Edition - Interactive Exploration and Analysis of Large-Scale Microbiome Sequencing Data

**DOI:** 10.1371/journal.pcbi.1004957

**Published:** 2016-06-21

**Authors:** Daniel H. Huson, Sina Beier, Isabell Flade, Anna Górska, Mohamed El-Hadidi, Suparna Mitra, Hans-Joachim Ruscheweyh, Rewati Tappu

**Affiliations:** 1 Center for Bioinformatics, University of Tübingen, Tübingen, Germany; 2 Life Sciences Institute, National University of Singapore, Singapore; 3 CeMeT GmbH, Tübingen, Germany; 4 IMPRS ‘From Molecules to Organisms’, MPI for Developmental Biology and University of Tübingen, Tübingen, Germany; 5 Norwich Medical School, University of East Anglia, Norwich, United Kingdom; Universite de Montreal, CANADA

## Abstract

There is increasing interest in employing shotgun sequencing, rather than amplicon sequencing, to analyze microbiome samples. Typical projects may involve hundreds of samples and billions of sequencing reads. The comparison of such samples against a protein reference database generates billions of alignments and the analysis of such data is computationally challenging. To address this, we have substantially rewritten and extended our widely-used microbiome analysis tool MEGAN so as to facilitate the interactive analysis of the taxonomic and functional content of very large microbiome datasets. Other new features include a functional classifier called InterPro2GO, gene-centric read assembly, principal coordinate analysis of taxonomy and function, and support for metadata. The new program is called MEGAN Community Edition (CE) and is open source. By integrating MEGAN CE with our high-throughput DNA-to-protein alignment tool DIAMOND and by providing a new program MeganServer that allows access to metagenome analysis files hosted on a server, we provide a straightforward, yet powerful and complete pipeline for the analysis of metagenome shotgun sequences. We illustrate how to perform a full-scale computational analysis of a metagenomic sequencing project, involving 12 samples and 800 million reads, in less than three days on a single server. All source code is available here: https://github.com/danielhuson/megan-ce

## Author Summary

Microbiome sequencing projects continue to grow rapidly, both in the number of samples considered and sequencing reads collected. With MEGAN Community Edition (CE), we provide a highly efficient program for interactive analysis and comparison of such data, allowing one to explore hundreds of samples and billions of reads. While taxonomic profiling is performed based on the NCBI taxonomy, we provide a number of different functional profiling approaches such as SEED, eggNOG, KEGG, and a new InterPro2GO classification scheme. MEGAN CE also supports the use of metadata in the context of principal coordinate analysis and clustering analysis.

This is a *PLOS Computational Biology* Software paper.

## Introduction

In microbiome analysis, 16S rRNA amplicon sequencing [1] is often used when a high-level analysis of taxonomic content suffices, and/or computational resources are limited. However, metagenomic shotgun sequencing allows a more detailed analysis of taxonomic composition and also provides a detailed functional analysis of a microbiome [2].

Individual samples usually involve millions of DNA reads and a typical project may involve hundreds of such samples [3]. An important step in the analysis of such data is the alignment of the reads against a protein reference database such as NCBI-nr [4] or InterPro [5]. The authors of [6] compared 250 million DNA reads from permafrost samples against the KEGG database [7] (containing less than 10 million sequences) using BLASTX [8] and this reportedly took 800000 CPU hours at a supercomputer center [9].

We recently published a new alignment tool called DIAMOND [10] that is able to align short metagenomic sequencing reads against the NCBI-nr database at 20000 times the speed of BLASTX without loss of sensitivity. This makes it possible to analyze large metagenome samples with little computational effort. For example, alignment of the permafrost data against the NCBI-nr database (containing over 60 million reference sequences) takes about one day on a single server with 32 cores.

Once all metagenomic reads of a sample have been aligned against a reference database, the next task is to then determine the taxonomic and functional content of the microbiome samples. This poses a number of computational challenges. First, how to process the large number of items (billions of reads and alignments) so as to support their efficient analysis? Second, how to provide user-friendly tools that allow interactive inspection and analysis of the data? Third, how to host and serve this data in a straightforward manner?

This paper addresses all three challenges, based on our new software MEGAN CE, which is a major rewrite and substantial extension of our MEGAN 4 metagenome analyzer tool [11]. This software performs taxonomic and functional analysis of reads. It also facilitates the interactive exploration and comparison of metagenomic samples. MEGAN uses a compressed, indexed file format (called RMA) to store reads, alignments, as well as taxonomic and functional classification information for a given sample. While such files can be produced interactively using MEGAN CE, we also provide a command line tool called blast2rma to compute such files on a server. Alternatively, in case that DIAMOND is used to compute alignments, we also provide a command line tool called Meganizer that can be run on a diamond file so as to perform taxonomic and functional binning of the reads in the file. The resulting information is appended to the file, together with additional indices required to efficiently access reads via taxonomic or functional classes. Meganized diamond files can be directly opened in MEGAN CE without any further processing and they are roughly the same size as the corresponding uncompressed fastq files.

Previous versions of MEGAN [11] required that files are present on the computer on which the software is running. While this remains possible with MEGAN CE, we also provide a new program called MeganServer (manuscript in preparation) that serves RMA and meganized diamond files over a local network or the internet.

**Fig 1:**
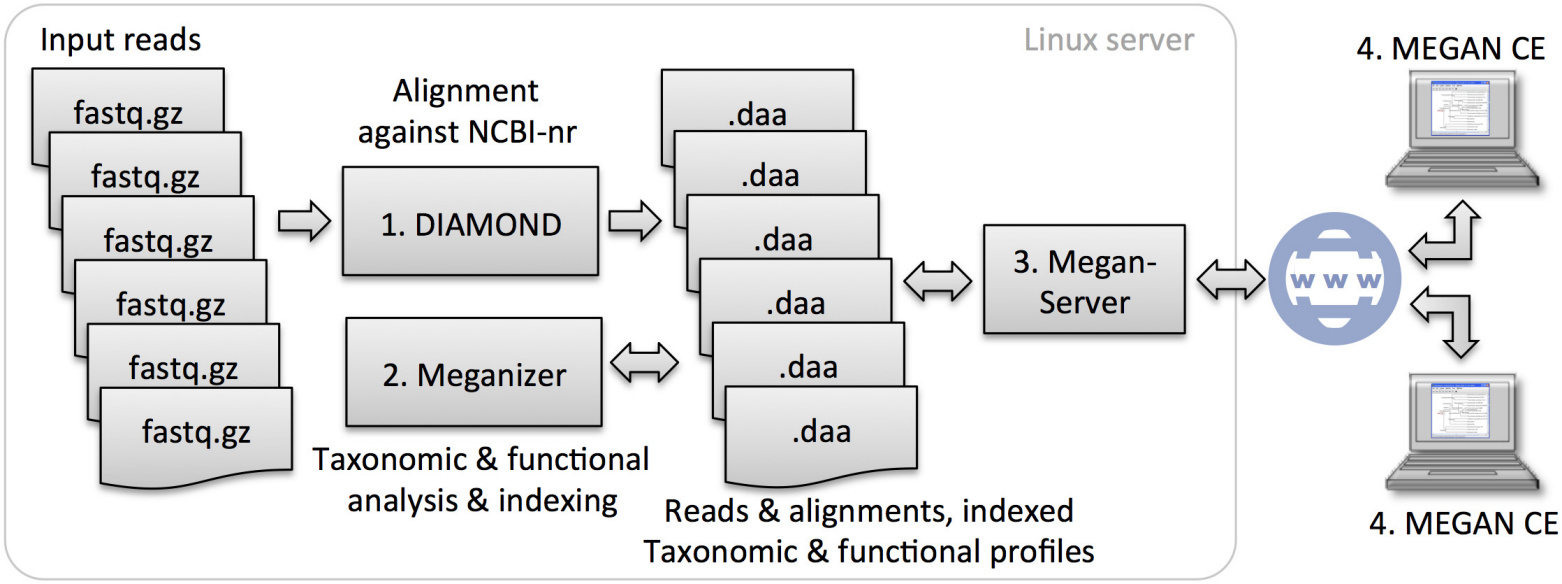
**The compressed fastq files in a metagenome or metatranscriptome sequencing project are (1) compared against a protein reference database such as NCBI-nr using DIAMOND.** (2) Taxonomic and functional analysis is then performed on the diamond files using Meganizer. (3) The resulting meganized diamond files remain on the server and are accessed via the MeganServer software. (4) Researchers work interactively with the data using MEGAN CE.

By integrating DIAMOND, MEGAN and MeganServer into a single, streamlined pipeline, we provide a straightforward and fast solution for microbiome analysis, facilitating the analysis of hundreds of samples and billions of reads on a single server in a matter of days. Any given sample is represented by only two or at most three files, namely the initial compressed fastq file obtained from a sequencer and either a meganized diamond file, when using DIAMOND, or an alignment file followed by an RMA file, when using some other alignment tool. In both cases, the resulting files contain all aligned reads, alignments and classification details. They can be stored on a server and made accessible through the MeganServer software, see Fig 1.

Two of the main goals of computational analysis of metagenomic data is to determine the taxonomic content of each sample, i.e. which organisms are present, and to estimate the functional capacity of the sample, i.e. which genes are present. This can be addressed by assigning sequencing reads to taxa and functional categories, based on their alignments to a reference database, in a process called binning.

By default, MEGAN CE performs taxonomic binning by assigning reads to nodes in the NCBI taxonomy using the LCA algorithm [12]. MEGAN CE supports a number of different classification systems for the binning of reads by function. A novel *InterPro2GO* analyzer uses a metagenome GO-slim [13] to classify InterPro families [5] and is based on files publicly available from EBI. MEGAN CE offers a SEED analyzer based on the concepts of subsystems and functional roles [14] and an *eggNOG* viewer based on the eggNOG extension of COGs [15]. In addition, MEGAN CE provides a legacy KEGG [7] viewer, based on files downloaded from KEGG in 2011.

One can easily execute principal coordinate analysis (PCoA) and cluster analysis using a number of different ecological indices and methods, and also compute standard alpha diversity indices.

In MEGAN CE, we offer a gene-centric approach to sequence assembly. The user can request to have all reads assigned to any given taxonomic or functional node assembled and output as contigs. This calculation is performed on-the-fly (manuscript in preparation) from within MEGAN, requiring no additional software or major calculations.

With DIAMOND, Meganizer, MeganServer and MEGAN CE, we provide a complete and highly-efficient solution for performing metagenome analysis. To illustrate the speed and sensitivity of our pipeline, we report on the computational analysis of a set of 12 human gut metagenomic samples, consisting of 800 million HiSeq reads [16]. From beginning-to-end, it took only 67 hours (wall-clock) on a single server, to align all reads against the NCBI-nr database (downloaded February 2015, approximately 64 million protein sequences) and then to perform taxonomic and functional analysis, using InterPro2GO, SEED, eggNOG and KEGG, involving 620 million reads and nearly ten billion alignments. MEGAN CE and MeganServer provide easy access to the resulting files, allowing users to perform both high-level analyses using trees, charts or PCoA plots, or low-level analyses such as drilling down to individual organisms, genes, reads or alignments, on single or multiple samples. This data can be accessed using MEGAN CE by opening the default public instance of MeganServer, which is hosted at the University of Tübingen.

Other popular standalone taxonomic analysis tools include MetaPhlAn [17], MetaPhyler [18] and Kraken [19]. Another is QIIME [20], which was initially developed to analyze 16S rRNA sequences. Services such as MG-RAST [21] and the EBI metagenomic web service [13] allow users to upload their data so as to use provided computational facilities for taxonomic and functional analysis of metagenomic sequencing data. See [22, 23] for two recent comparisons of the performance of different approaches.

## Design and Implementation

This paper introduces MEGAN Community Edition (CE), which is a major update of our MEGAN software [11]. This release contains a large number of new features and has been substantially rewritten so as to support the analysis of many samples (hundreds) and many reads (billions). This release includes a number of command line tools, in particular blast2rma, daa2rma and Meganizer, which can all be used to prepare input files for MEGAN CE.

In addition, we recommend the use of DIAMOND [10] for ultra-fast alignment of reads against NCBI-nr and MeganServer to allow web access to MEGAN files.

### RMA files

MEGAN CE analysis requires that sequencing reads are first aligned against a suitable reference database, such as NCBI-nr in the case of a protein-based analysis, or Genbank for a DNA-based analysis, or the Silva database [24], say, when aligning 16S rRNA reads. MEGAN CE can import reads and alignments in a number of different file formats and computes a compressed and indexed binary file in so-called RMA format that contains all reads, alignments, taxonomic and functional classifications. The file is indexed to allow quick access to reads and alignments by taxonomic or functional assignment. MEGAN CE also provides a command line program called blast2rma for computing RMA files from BLAST-like alignments. With MEGAN CE, we introduce a new version of the RMA format that requires much less disk space than previous versions.

### Meganizer

Our alignment program DIAMOND produces “diamond files” in a binary output format called DAA (“Diamond alignment archive”), from which both tabular and SAM format can be extracted. We provide a new program called Meganizer that analyses all reads present in a given diamond file, performs taxonomic and functional analysis of them, and then appends the resulting classifications and indices to the end of the diamond file. Meganizing a diamond file takes much less time than generating an RMA file and reduces the number of files that are created during metagenome analysis. Indeed, using DIAMOND and Meganizer, each sample in a metagenome study is represented by only two files, namely the original compressed fastq file and the resulting meganized diamond file. This file is usually smaller than the corresponding RMA file.

### Taxonomic assignment

By default, MEGAN CE uses the naive LCA algorithm [12] to perform *taxonomic binning*. In this approach, each read is assigned to the “lowest common ancestor” node in the taxonomy that lies above all species for which the read has a significant alignment. The rationale here is that reads that align to widely conserved genes should be assigned to high-level taxa (such as the rank of Phylum), whereas reads that align to a gene that is specific to a given type of organisms should be assigned to a more lower taxon (such as at the rank of Genus or Species). As a consequence, reads are binned across all taxonomic ranks.

The naive LCA algorithm provides a conceptually straight-forward and fast approach to taxonomic binning, running at a rate of over 100 million reads and 2 billion alignments per hour on a single server, as discussed below. However, it is less suited for purposes of taxonomic *profiling*, where the goal is to obtain an accurate estimation of the taxonomic content of a sample, see [22, 23]. One reason for the poorer performance of the naive LCA algorithm as a profiling tool is that it processes each read in isolation, independent of all other reads.

To address this issue, in MEGAN CE we provide an implementation of the *weighted LCA* algorithm, which was developed in the context of the 2013 DTRA Algorithms Challenge [25, 26]. The weighted LCA algorithm operates as follows. In a first phase, each reference sequence *S* is assigned a weight. This is the number of reads *R* that only align to *S* (or to other references as well, as long as they have the same species assignment as *S*). Then, in a second phase, each read *R* is placed on the lowest node in the taxonomy that is above 75% (by default) or more of the total weight of all references to which *R* has a significant alignment. This improves the specificity of taxonomic assignment, but requires more time to run.

A *taxonomic profile* is usually calculated at a single specific taxonomic rank *H* and aims at providing of the number of reads attributable to each of the taxa at the given rank (such as a Genus-level profile). In contrast, both the naive and the weighted LCA algorithm assign reads across all ranks of the taxonomy. To summarize all counts at a fixed taxonomic rank, MEGAN CE provides a simple *projection algorithm*, which operates as follows. First, all reads that are assigned by the LCA algorithm to some taxon node *t* that lies *above* the desired rank *H* are pushed down to the children of *t* in proportion to the number of reads assigned on or below each of the the children. This is repeated until all reads have been pushed down to a node that lies in *H*. Second, all reads that are assigned by the LCA algorithm to some taxon node *below* the desired rank *H* are simply assigned to the ancestor node that lies in *H*.

### InterPro2GO viewer

**Fig 2:**
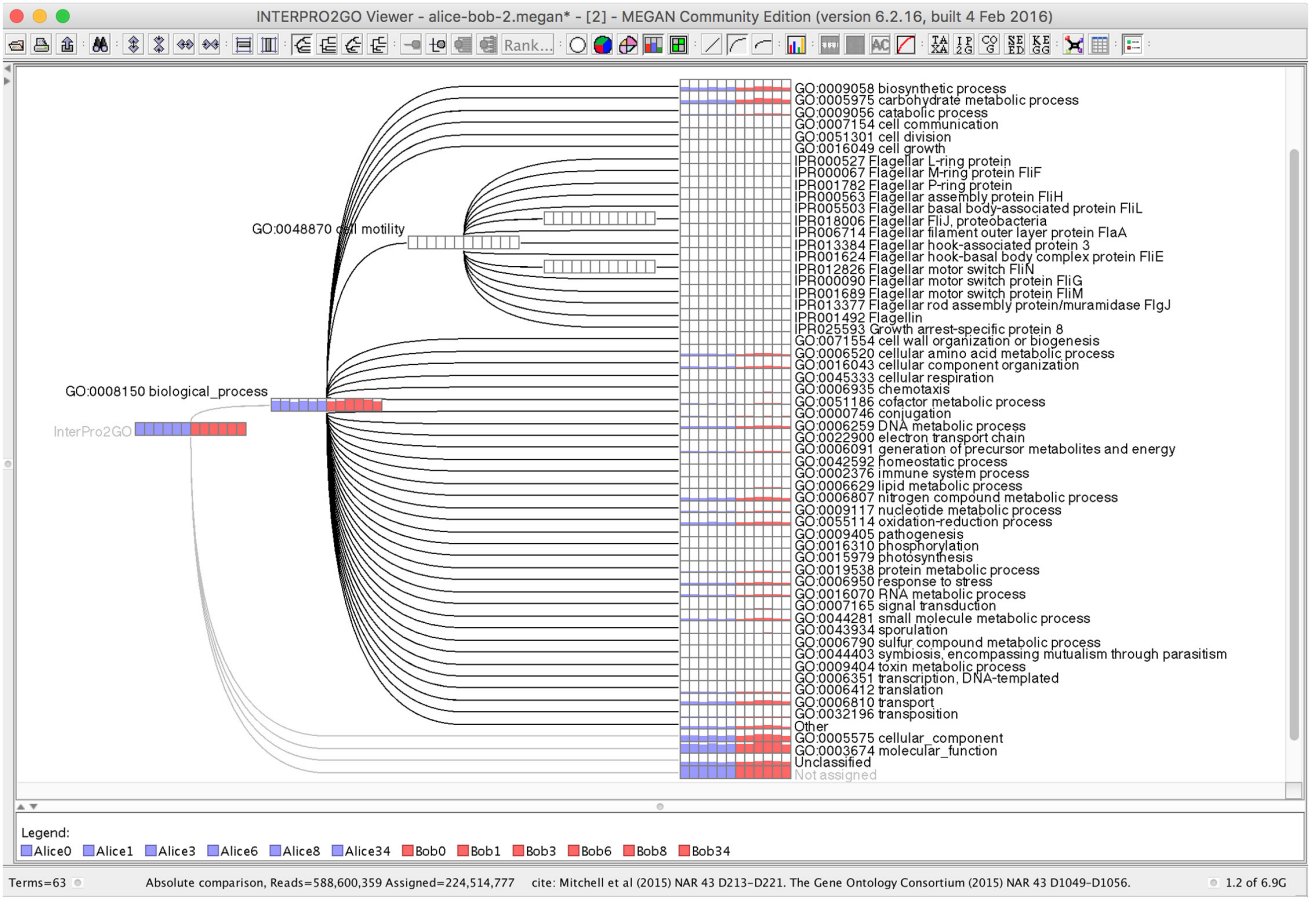
The new InterPro2Go viewer. High-level nodes represent the metagenomic GO-slim [13], whereas low-level nodes are based on InterPro [5]. Here we have uncollapsed the GO “biological process” domain node to show the second tier nodes attached below it. Each node is labeled by a bar chart representing the number of reads assigned to the node, or below it, for 12 different human stool samples [16]. In this example, 27.5% of 816 million reads are assigned to an InterPro family by MEGAN CE.

The EBI metagenome service provide a hierarchical classification of reads by Gene Ontology assignment based on the metagenomic GO-slim [13] and a tabular mapping of reads to InterPro families [5]. Based on these concepts and data downloaded from http://www.ebi.ac.uk/interpro and http://www.uniprot.org, we have designed a novel InterPro2GO hierarchical viewer in which the top two tiers of nodes are based on the metagenomic GO-slim and all lower-level nodes represent InterPro families (see Fig 2).

Our new InterPro2GO classification has three first-tier nodes labeled “GO:0008150 biological process”, “GO:0005575 cellular component” and “GO:0003674 molecular function”, which represent the three domains of the Gene Ontology [27]. Below these, there are 84 second-tier nodes that provide a refined GO-based classification of function. A third tier of nodes represents all InterPro families that have an assignment to one or more GO categories. An InterPro family will give rise to more than one node in the graph, if it maps to different GO domains. An InterPro family that maps to multiple second-tier GO nodes in the same GO domain is placed below an “Other” node that is associated with the GO domain node. By construction, the resulting classification is a tree in which each InterPro family occurs up to three times, at most once below each domain node.

A fourth first-tier node labeled “Unclassified” is a catch-all node for all InterPro families that have not been assigned a GO assignment. The InterPro2GO classification in MEGAN CE has approximately 26 000 nodes in total.

### SEED viewer

The SEED [14] is a functional classification that is based on an assignment of genes to “functional roles”. These are grouped into “subsystems” of related functional roles that make up a metabolic pathway, a complex, or a class of proteins. MEGAN CE uses a significantly updated SEED viewer that is based on files downloaded from SEED in November 2015. Reads are binned to functional roles.

### eggNOG viewer

MEGAN CE offers a new eggNOG viewer that is based on a classification of orthologous groups in which reads are binned to “clusters of orthologous groups” (COGs) and “non-supervised orthologous groups” that appear as leaves of the eggNOG classification [15].

### KEGG viewer

MEGAN CE also provides a functional viewer based on KEGG [7]. Here, KEGG othologous groups (KO groups) are mapped to enzymes that appear in metabolic pathways. MEGAN CE ships with a legacy representation of KEGG that is based on files downloaded from the KEGG website in early 2011. For users in possession of a KEGG ftp license, a separate program called MEGAN UE (Ultimate Edition) provides tools for generating an up-to-date representation of KEGG in MEGAN UE.

### Mapping reference sequences

The classification of metagenomic reads depends on a classification of the reference sequences to which they align. MEGAN provides three mechanisms for determining the classification identity of reference sequences. First, MEGAN can scan for a classification tag in the header line of a reference. For example, a NCBI taxon id may be written as tax|666. Second, MEGAN supports the mapping of GI numbers to taxon or functional identifiers using a file-based index. Third, MEGAN supports the mapping of alpha-numerical accession numbers to taxonomic or functional identifiers using a file-based hash-table, in anticipation of NCBI’s plan to discontinue the use of GI numbers.

### Working with multiple samples and metadata

Any number of metagenome samples can be opened together in a single “comparison document”, using a dialog that allows one to source samples both from local disk and from any instance of MeganServer that MEGAN CE is currently connected to.

**Fig 3:**
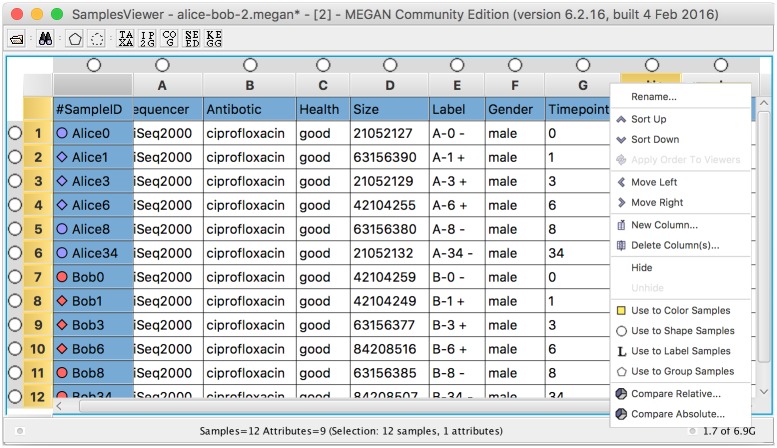
Spreadsheet for entry and analysis of metadata associated with samples.

Metadata such as technical and clinical parameters, or physical and bio-chemical measurements, can be attached to individual samples. When such samples are combined into a single comparison document, then all associated metadata is merged into a single table. To facilitate working with metadata, MEGAN CE provides an interactive “sample viewer” that is based on a versatile spreadsheet that can be used to add or edit metadata, and also to select or group samples by metadata attribute values, or to set colors, labels, shapes by such values (see Fig 3). Metadata can be imported or exported using a standard CSV format that is compatible with other metagenome analysis tools.

Given a comparison document containing multiple samples, the sample viewer also allows one to extract individual samples, or to compute the “total biome” (i.e. the union of all samples) or the “core biome” (i.e. those taxa that appear in a given minimum percentage of all samples). One can also easily merge and compare samples based on the values of a selected attribute.

### PCoA, bi-plots and tri-plots

**Fig 4:**
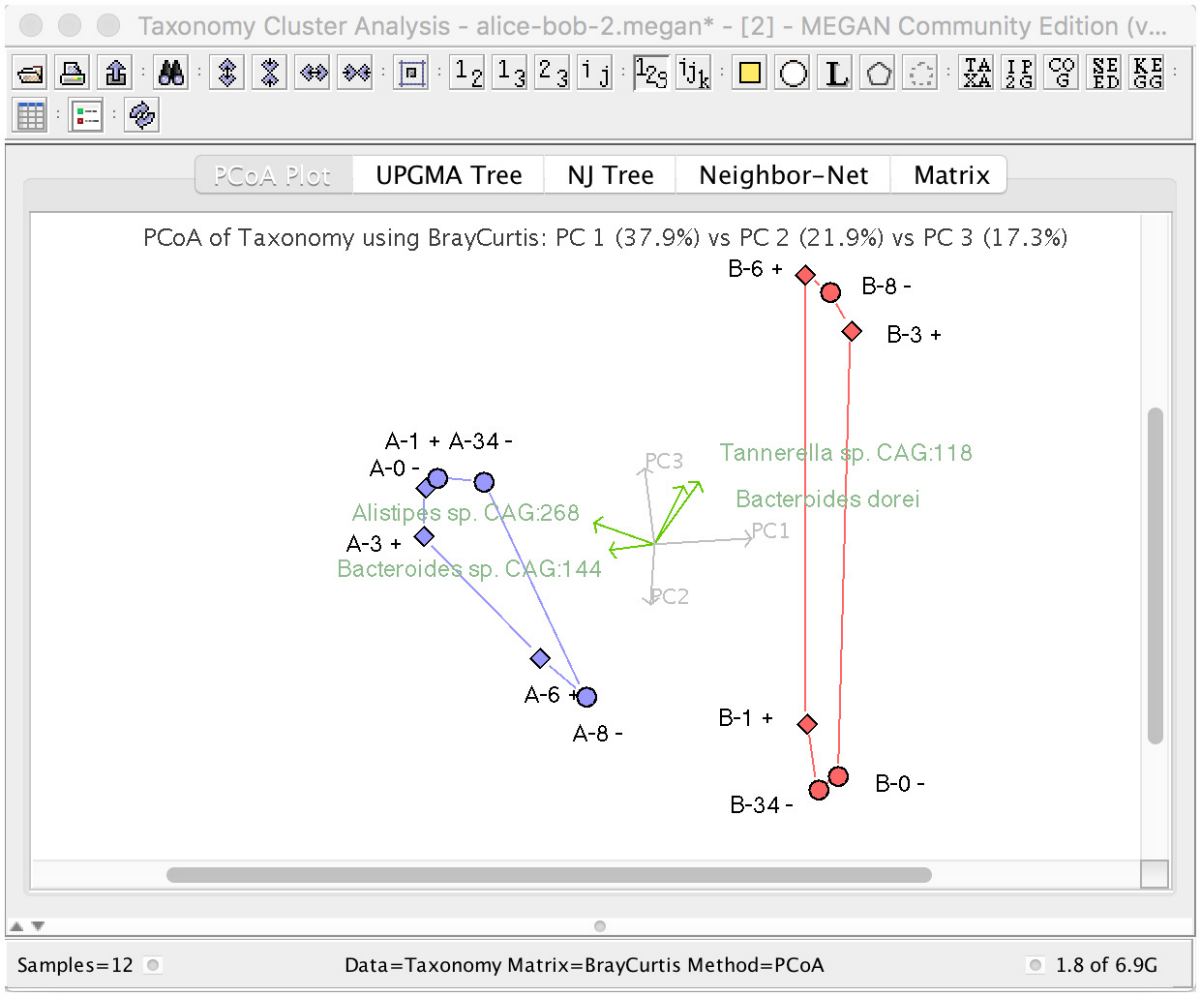
A PCoA analysis of 12 human gut samples [16] computed using species-level profiles and Bray-Curtis distances. Samples are labeled by subject pseudonym, day 0–34 and whether antibiotics were taken (+) or not (-) on the given day. For both subjects, the plot clearly shows that the taxonomic profiles move further and further away from the original during the course of antibiotics, but then return back close to the original at the end of the study. The top five bi-vectors are also shown, labeled by species name.

PCoA (principle coordinate analysis) is a standard tool used in the analysis of microbiome data [28]. MEGAN CE allows the user to perform such analysis in two or three dimensions, based on taxonomic or functional profiles. The program offers a number of different ecological indices to be used in the calculation of PCoA plots, such as the euclidean distance, in which case the resulting plot is identical to the result of a PCA (principle component analysis), the Bray-Curtis distance [29], or the Jensen-Shannon distance, to name a few. MEGAN CE provides an implementation of a bi-plot, in which the taxa or functional groups that contribute the most to the variation shown in the PCoA plot are represented by vectors that indicate the direction of steepest increase (see Fig 4). MEGAN CE also provides a tri-plot, in which metadata that correlates the strongest with the changes shown in the PCoA plot are indicated by vectors.

### Gene centric assembly

The aim of metagenome assembly is to stitch sequencing reads together so as to obtain longer stretches of contiguous sequence (contigs) of the genomes of the organisms present in a given microbiome. Metagenome assembly from Illumina reads is considered a difficult problem [30], although progress is being made [31]. In the past, one main reason for performing metagenome assembly has been to reduce the total amount of sequence that has to be aligned against a reference database, while another reason is so as to improve specificity. However, the introduction of DIAMOND has changed this equation and aligning all reads without an initial assembly step is now usually the faster option.

In MEGAN CE, we provide a gene-centric approach to sequence assembly that aims at assembling individual genes at the strain level, guided by protein alignments (see also e.g. [32]). The program can assemble all reads assigned to a specific node in a taxonomic or functional classification, allowing one to investigate the sequence variability of a given gene. This calculation is triggered interactively and is performed on-the-fly. Protein alignments to reference sequences are employed to infer DNA overlaps between reads, giving rise to an overlap graph, from which contigs are extracted (manuscript in preparation).

## Results

We illustrate the application of DIAMOND, Meganizer, MEGAN CE and MeganServer using public data from a recent study that we were involved in [16]. In this study, two healthy volunteers, with aliases Alice and Bob (although both are males) were administered a course of antibiotics (ciprofloxacin, days 2–6). Stool samples were collected on days 0, 1, 3, 6, 8 and 34, resulting in a total of 12 samples. The samples were subjected to shotgun metagenome sequencing (HiSeq2000, paired end, 100bp), resulting in 816 million reads (see Table 1).

**Table 1:** For twelve shotgun metagenome samples [16], we report (a) the number of reads, (b) wall-clock time required to align the reads against NCBI-nr using DIAMOND, (c) the number of matches obtained, (d) the number of reads that have at least one alignment and (e) the time required to run Meganizer to perform taxonomic and functional classification of all reads. The total wall-clock time is 67 hours on a single server with 32 cores.

	(a)	(b)	(c)	(d)	(e)
Sample	Reads	DIAMOND (s)	Alignments	Aligned reads	Meganizer (s)
Alice 0	66 393 401	19 062	627 405 772	44 900 227	9 299
Alice 1	64 923 975	15 771	595 715 349	43 498 105	11 338
Alice 3	55 092 349	13 435	515 249 349	37 675 494	8 621
Alice 6	66 289 376	16 801	910 892 059	52 627 776	11 771
Alice 8	57 957 661	14 134	790 946 244	45 358 448	13 911
Alice 34	64 380 386	15 615	608 114 143	44 741 897	11 962
Bob 0	61 232 588	14 573	825 213 917	48 882 884	12 058
Bob 1	65 763 766	16 203	841 038 616	51 408 892	12 270
Bob 3	89 034 641	34 598	1 233 571 041	72 017 720	15 789
Bob 6	89 339 172	27 333	1 138 796 522	70 344 161	15 507
Bob 8	78 001 118	19 734	1 049 831 855	63 336 241	13 423
Bob 34	57 627 119	15 406	780 844 319	45 568 158	11 433
Total	816 035 552	222 665	9 917 619 186	620 360 003	Max: 15 789
Time		≈ 62 h			≈ 5 h

The wall-clock time for the complete taxonomic and functional analysis of this data was 67 hours on a single server (32 cores, 512GB of memory). In more detail, DIAMOND alignment of all 816 million reads against NCBI-nr (downloaded February 2015) took 62 hours (wall-clock time), resulting in just under ten billion alignments. Meganization of the resulting diamond files took an additional 5 hours, running Meganizer in parallel on all 12 samples. The fastq files have a total (uncompressed) size of 199 Gb, whereas the meganized diamond files occupy 150 Gb. Loading the files into MeganServer took about one minute.

DIAMOND found one or more significant alignments against NCBI-nr for 75% (620 million) of the 816 million input sequencing reads. Taxonomic analysis assigned 71.7% (585 million) to a taxon. Functional analysis using InterPro2GO assigned 27.5% (224 million) to an InterPro family. Functional analysis using SEED assigned 10% (82 million) to a functional role in SEED. Functional analysis using eggNOG assigned 17.5% (134 million) to a COG or eNOG. Functional analysis using KEGG (downloaded July 2015) assigned 14% (114 million) of the total reads to a KO.

While the main focus of the study reported by [16] was to determine how the levels of antibiotic resistance genes change in the gut during the course of antibiotic treatment, in Fig 4 we present a PCoA analysis of all samples, computed using Bray-Curtis distances. For both subjects, we clearly see that the taxonomic profiles of their stool samples move away from the original as the treatment progresses. On day 34, that is, 28 days after the end of the course of antibiotics, in the case of Bob, the taxonomic profile is practically indistinguishable from his original profile, while in the case of Alice it has returned most of the way.

We also display the top five bi-plot vectors. The vectors point in the direction of those samples that have substantially higher reads counts for the given species. For example, *Bacteroides stercoris CAG:120* points in the direction of samples A-6 and A-8, whereas the *Bacteroides dorei* vector is oriented toward B-3, B-6 and B-8. Interestingly, much of the clear separation of Alice’s and Bob’s samples is based on Bacteroides species, which is a difference that would be lost in a Genus-rank comparison.

## Availability and Future Directions

The programs MEGAN CE, DIAMOND and MeganServer can be downloaded here: http://www-ab.informatik.uni-tuebingen.de/software.

MEGAN Community Edition is free software under the GNU General Public License. All source code is available here: https://github.com/danielhuson/megan-ce. User support for the Community Edition is provided through a community website at http://megan.informatik.uni-tuebingen.de.

With MEGAN CE, DIAMOND, Meganizer and MeganServer, we provide a powerful suite of programs that allow researchers to explore and analyze microbiome sequencing samples interactively on a very large scale. Future work will continue to focus on speeding up the analysis and supporting the exploration of ever greater numbers of ever larger microbiome sequencing samples, fueled by the continuing decrease in the price of sequencing.
